# Editorial

**Published:** 1903-03-15

**Authors:** 


### EDITORIAL.

A BILL has been introduced into the Illinois Legislature for the enactment of a new dental law. In the main the new law is good. But it contains two features which are somewhat inconsistent, and which will probably give future examiners no little trouble. One of these objectionable features is to make the examining board a kind of board of censors, with the privilege of inspecting dental colleges and deciding as to whether they are doing their work in accordance with the ideals of the examiners. At the same time the colleges are given no recognition for the work whether done poorly or excellently. That is to say, the college graduate has no better standing before the board than the applicant for registration who presents himself without ever having had any systematic training of any sort. Every one must take the examination regardless of his previous training, which evidently is accounted in the case of the college man as only pupilage. It would seem as though a board which dictates the teaching standard of a school ought to be willing to credit its product. In section 9 of this law its last clause, states in substance that licenses may be revoked which have been issued to men who may afterwards engage in unprofessional conduct. We do not think such a power can be given to any State officials, as it is retroactive. In the hands of a good board such a law would doubtless have great advantages for its restraining influence; but its enforcement will doubtless bring about trouble and probable annulment of the entire statute.

---

THE Odontographic Society of Chicago has just celebrated its tenth anniversary by holding one of the largest dental meetings ever held in this or any other country. It is stated that more than twenty-five hundred dentists were present, and registered, and that they came from every part of the country. About one hundred and thirty clinics and five essays made up the program. A large number of exhibits also helped to increase the attractiveness of the gathering. It was truly a great meeting and is characteristic of Chicago. Everything in Chicago is is gauged by its "bigness." We do not mean by this that they do not have other standards, but they seem to appreciate things in proportion to their size more there than in Boston, or Philadelphia, for instance. The papers and clinics were reported of the highest order and no doubt much good will come from this meeting, in spite of the fact that it was practically impossible for any one to see the exhibits or witness the clinics with much comfort. The reading and discussion of papers before such an audience is not very satisfactory to the essayist or listeners. It was, nevertheless, a remarkable gathering as it represented nearly one-tenth of the total number of practitioners in the entire country, and is twice as large as the Columbian Dental Congress, which was before the largest meeting ever held in the country. and was internationally represented. This meeting demonstrates that it is possible to attract the members of our profession to a meeting which promises to bring them in contact with up-to-date men and methods, and will serve as a good hint to be followed up by local societies in working up their programs and drawing out their constituents. It also illustrates what a few enterprising and zealous men can do when they work hard and harmoniously. Such events engender enthusiasm and inspiration.

—✦—

At the recent meeting in Chicago it was announced that Harvard University Dental School would not take up the four years course next fall, because it contemplated in the near future requiring an academic degree for admission to its course. This is unfortunate for Harvard and the cause of education as well. It has been a hard struggle to get the profession and especially the teaching part of it to feel that it was expedient to lengthen the time of the course, although all teachers are agreed that the time is necessary to prepare men for up-to-date practice. The reason given by Harvard for not beginning the course is good, providing their school makes the advance in entrance requirements next fall. But we see no reason why a year of advanced credit should not be allowed a man entering on an academic degree just as a year of credit is allowed for graduate medical work. We are confident the Faculties Association will grant such concession, and there need not then be any cause for a break in the solidarity of the teaching forces of our country. If the expressions of the representative teachers present at Chicago when the matter was discussed can be relied upon as expressing the sentiment of all other teachers, there will be no backward steps taken in this matter, and every effort will be made to induce Harvard authorities to co-operate in what will undoubtedly be one of the best moves in educational affairs ever made.

—✦—

THE *International Journal* contains in its February issue an article on "Regeneration of the Pulp", which sets up a most heretical doctrine of the probable regeneration of the pulp after surgical extirpation, to the extent of filling the pulp canal with new pulp tissue. It is what might be termed in Chinatown, merely a "pipe dream". It is possible that under right conditions there might be a proliferation of connective tissue, cells and blood vessels, but no pulp organ with its odontoblast layer is credible.

